# Ex Vivo Confocal Microscopy Speeds up Surgical Margin Control of Re-Excised Skin Tumors and Greatly Shortens In-Hospital Stay

**DOI:** 10.3390/cancers16183209

**Published:** 2024-09-20

**Authors:** Frank Friedrich Gellrich, Jörg Laske, Julian Steininger, Nadia Eberl, Friedegund Meier, Stefan Beissert, Sarah Hobelsberger

**Affiliations:** 1 Department of Dermatology, Faculty of Medicine, University Hospital Carl Gustav Carus, 01307 Dresden, Germany; joerg.laske@ukdd.de (J.L.); julian.steininger@ukdd.de (J.S.); nadia.eberl@ukdd.de (N.E.); friedegund.meier@ukdd.de (F.M.); stefan.beissert@ukdd.de (S.B.); sarah.hobelsberger@ukdd.de (S.H.); 2 Carl Gustav Carus Faculty of Medicine, Technische Universität Dresden, Fetscherstraße 74, 01307 Dresden, Germany; 3 Skin Cancer Center at the University Cancer Center, 01307 Dresden, Germany; 4 National Center for Tumor Diseases (NCT/UCC), 01307 Dresden, Germany; 5 German Cancer Research Center (DKFZ), 69120 Heidelberg, Germany; 6 Helmholtz-Zentrum Dresden-Rossendorf (HZDR), 01328 Dresden, Germany

**Keywords:** reflectance confocal microscopy, non-melanoma skin cancer, margin control, 3D histology, dermatologic surgery

## Abstract

**Simple Summary:**

This research explored a new method to evaluate surgical margins when removing non-melanoma skin cancer, aiming to reduce the time patients need to stay in the hospital. Typically, surgeons use 3D histology to ensure all cancer tissues have been removed, but this process is time-consuming. This study suggests using ex vivo reflectance confocal microscopy (evRCM), which allows for quicker margin assessments. While evRCM showed high accuracy in confirming cancer-free areas, it was not as effective in detecting small remaining cancer cells as histology. Despite this limitation, evRCM significantly reduced patients’ time in the hospital. These findings suggest that evRCM could become a valuable tool for improving the speed and accuracy of skin cancer surgery; however, its effectiveness is limited. Close collaboration between surgeons and pathologists is mandatory to achieve good patient outcomes.

**Abstract:**

Background/Objectives: To ensure that non-melanoma skin cancer (NMSC) is completely removed in healthy tissue, micrographically controlled surgery (3D histology) is often performed, which can prolong the inpatient stay. This study examined ex vivo reflectance confocal microscopy (evRCM) for perioperative assessment of surgical margins, specifically in cases where re-excision was necessary due to incomplete removal of cutaneous tumor tissue. Methods: NMSC re-excisions were evaluated using evRCM by a cutaneous surgeon, with retrospective review by an independent pathologist when results differed from histology. Results: evRCM demonstrated high specificity (0.96; 95% CI, 0.90–0.99) but low sensitivity (0.20; 95% CI, 0.06–0.51). Unlike pathology, which discards outer surgical margins, evRCM examined the true surgical margins. Retrospective pathology analysis of the misdiagnosed cases confirmed that 25% (*n* = 2/8) were false negative and 75% (*n* = 6/8) were potentially false positive, resulting in a sensitivity of 0.2–0.8. Notably, evRCM led to a 113-day reduction in in-hospital stays, probably resulting in increased patient satisfaction and cost-effectiveness. Conclusions: evRCM was valuable for speeding up the assessment of surgical margins in patients with re-excised NMSC. Proper tissue preparation and assessment require interdisciplinary collaboration between cutaneous surgeons, pathologists, and physician assistants, emphasizing the need for standardized operating procedures.

## 1. Introduction

Non-melanoma skin cancer (NMSC) is the most common tumor type in people with light skin and has shown a steady increase in incidence over the last few decades [1,2,3,4,5,6,7]. Basal cell carcinoma (BCC) is the most common NMSC [8], accounting for 75% of all keratinocyte cancers [9]. Squamous cell carcinoma (SCC) is the second most common NMSC [10], with a share of 20% [11]. Most NMSCs can be treated curatively by radical excision or various local therapies [12]. Excision is performed either by a standard excision (2D) with standardized safety margins or by a micrographically controlled surgery (3D) with mapping of the circumferential and deep tumor borders and subsequent step-wise re-excisions in case of any tumor remnants [9,13,14]. After excision, a histological assessment of the margins is recommended. In standard excision (2D), the tumor is processed in multiple cross-sections, known as the bread loaf technique [15]. However, tumor remnants might be overlooked in the lateral margins between cross-sections. Micrographically controlled surgery should be performed in cases with high-risk factors, BCC, and SCC [16]. Both cancers show lower recurrence rates when micrographically controlled surgery is performed [17,18]. In Europe, two techniques for the complete circumferential and deep-margin processing of tissue specimens have been established. In Mohs micrographic surgery (MMS), intra-operative frozen sections are assessed [19,20]; in 3D histology, paraffin sections are assessed [19,20], usually requiring patient hospitalization to perform the excision and temporarily cover the defect while preparing the tissue sections overnight to be analyzed by a pathologist the next morning. Depending on the findings, the defect is closed if the surgical margins are clear (R0) or re-excised if tumor remnants in the surgical margins are found (R1), temporarily covering the defect again pending re-assessment. Each re-excision prolongs the patient′s hospital stay, causing complications (infections, loss of mobility in older adults, etc.), higher healthcare costs, and difficulty in planning the daily operating schedule.

The ex vivo Reflectance Confocal Microscopy (evRCM) technique enables an immediate perioperative assessment of excised tissue margins [21,22]. RCM allows for skin assessment at a cellular resolution down to a depth of approximately 200 µm [22] and has been used for more than ten years for in vivo diagnosis and margin estimation [23] of melanomas, BCCs, and SCCs. RCM shows high sensitivities and specificities for diagnosing BCC and SCC [24,25]. evRCM uses the same technique but allows for perioperative examination of excised tissues. The excised tissue is immersed for a few seconds in two fluorescent dyes, distilled water, and alcohol, fixed between two glass discs, and scanned from the bottom side within seconds to a few minutes, depending on its size. Unlike conventional RCM, evRCM uses a second laser with a different wavelength to scan cell nuclei and membranes using the fluorescent dyes. After digital staining, an image resembling a hematoxylin-eosin (HE)-stained histology section is generated.

evRCM showed promising results for real-time assessment of benign and malignant skin tumors with a high concordance with histological findings [21,26,27,28,29,30,31,32,33]. It has also been used with good results to assess surgical margins of BCCs [28,34] and various solid tumor types, including breast, prostate, central nervous system, kidney, bladder, and conjunctival tumors [35]. evRCM has not yet become widely accepted for surgical margin assessment of NMSCs, as detecting small tumor areas, such as perineural growth of SCC, is sometimes difficult [13,36]. Moreover, scanning skin tissue with evRCM poses a particular challenge due to the difficulty of visualizing the epidermis.

Like classic RCM, evRCM images can be assessed by a dermatologist rather than a pathologist. This study used evRCM to examine perioperative re-excisions of patients with previous incomplete NMSC excisions to facilitate decisions on immediate re-excision or defect closure and shorten the hospital stay. The evRCM images were analyzed by the cutaneous surgeon who performed the surgery. The study aimed to compare the effectiveness of evRCM findings to histology and assess the impact on the patient′s medical treatment.

## 2. Materials and Methods

This prospective, single-center study included 102 excisions in 98 patients performed between 26 May 2023 and 6 August 2024 at the University Hospital Carl Gustav Carus in Dresden, Germany. The Ethics Committee at TU Dresden approved the study protocol, which complied with the Declaration of Helsinki (BO-EK-176052024). The data were recorded as part of the normal documentation during inpatient treatments and were analyzed anonymously without the patient’s written consent following §29 (3) of the Saxon Hospital Act and the decision of the Ethics Committee.

Patients with an indication for inpatient excision of NMSC were usually treated at the University Hospital Carl Gustav Carus in Dresden using the slow Mohs surgery. After tumor excision on the first day of surgery, the defect was temporarily covered, and the tissue was sent to the pathology department for processing and histological excision margin analysis the next morning. In cases of incomplete excisions, a re-excision was performed on the second day of surgery with a perioperative examination of the surgical margins using evRCM. If a complete excision was determined, the defect was closed directly; if an incomplete excision was detected, a further re-excision was made. In cases with unclear findings, the procedure proceeded based on the surgeon’s choice. After diagnosis using evRCM, the tissue was examined histologically the following morning. The patients remained in the hospital a further night after defect closure for monitoring and were discharged the next morning, depending on the final histological findings.

**Figure 1:**
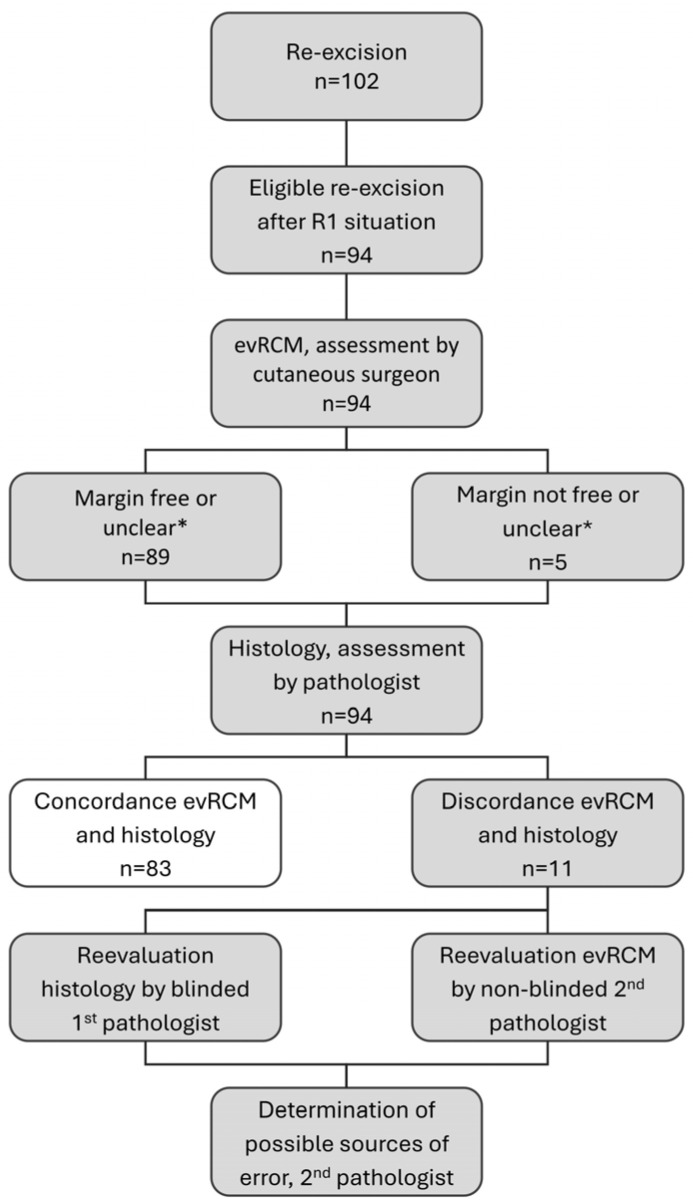
Study procedure; * during the analysis, evRCM findings assessed as unclear were evaluated according to their surgical consequence. Concordant findings in evRCM and histology (white colour) were not re-evaluated.

The evRCM assessment was performed by cutaneous surgeons with extensive experience in various in vivo imaging techniques, but without histological qualifications. An independent, blinded pathologist retrospectively assessed histological findings that differed from the evRCM assessment. Subsequently, a second, unblinded pathologist assessed the corresponding evRCM images in conjunction with the histological findings to determine possible sources of error, the diagnostic confidence of evRCM, and epidermis visibility using evRCM (Figure 1).

We used the VivaScope 2500 (VivaScope GmbH, Munich, Germany) to acquire confocal microscope images.

Statistical analysis was performed using IBM SPSS Statistics for Windows, Version 28.0 (IBM Corp., Armonk, NY, USA).

## 3. Results

This study included 102 excisions of NMSC whose surgical margins were assessed by evRCM. Seven cases were excluded because evRCM was used to assess the surgical margins of a primary excision rather than a re-excision, and one case was excluded due to incorrect documentation. Consequently, we analyzed 94 excisions from 90 patients.

The patient age was 78.5 ± 10.3 (38–94) years, and there were 47 males (52.2%) and 43 females (47.8%).

Among the tumors, 92 were in the head and neck region, 1 on the trunk, and 1 on a limb. Within the head and neck region, 32 tumors were located on the top of the head, 27 on the nose, 8 on the ear, 2 on the neck, 1 on the lips, and 22 on other parts of the face.

### 3.1. Histological Findings of the Primary Excision

The tumors included 82 (87.2%) BCCs, 11 (11.7%) SCCs, and 1 (1.1%) Bowen’s disease. Eighty-nine tumors were re-excised laterally, four basally, and one laterally and basally. All were examined by evRCM.

The BCCs were assigned one or two histological subtypes and included 50 nodular, 21 sclerodermiform, 14 superficial, 9 micronodular, 4 cystic, and 1 metatypical subtype. The BCC tumor thickness was 2.1 ± 1.4 (0.3–6.2) mm with an infiltration level of 4.1 ± 0.4 (2–5). Ulceration was present in 22 (23.4%) BCCs.

The SCC tumor thickness was 3.0 ± 2.0 (1.5–6.5) mm with an infiltration level of 4.5 ± 0.5 (4–5). One SCC was highly differentiated (G1), nine were moderately differentiated (G2), and one was poorly differentiated (G3). Ulceration was present in three (27.3%) SCCs, and one showed perineural sheath invasion (Table 1).

### 3.2. evRCM and Histological Findings Following Re-Excision

Using evRCM, 88 (93.6%) of the re-excisions were assessed as tumor-free (R0), 3 (3.2%) with tumor residuals, and 3 (3.2%) as unclear. Consequently, a re-excision was performed in 4 (4.3%) cases. The defect was closed in the same surgical session in 89 (94.7%) cases and was temporarily covered to await the histological findings in 5 (5.3%) cases. Two of the three cases with unclear findings were re-resected, and one was closed directly. When calculating the sensitivity and specificity, these cases were evaluated according to their consequence.

The histological evaluation revealed an incomplete excision in the surgical margin of 10 (10.6%) re-excisions, 9 BCCs, and 1 SCC. The evRCM findings matched the histological ones in 83 (88.3%) cases, detecting 81 (96,4%) complete and 2 (20.0%) incomplete excisions. Eight histologically incomplete excisions were evaluated as complete by evRCM, and three histologically complete excisions were evaluated as incomplete by evRCM. Among these 11 discordant findings, 3 were assessed as unclear. Based on the histological findings, the sensitivity of evRCM was 0.20 (95% CI, 0.06–0.51), the specificity was 0.96 (95% CI, 0.90–0.99), and the diagnostic accuracy was 0.88 (95% CI, 0.80–0.93; Table 2).

Eleven discordant histological and evRCM findings were retrospectively assessed by a pathologist. In one additional case, a complete excision was evident on the outermost section of the histological slide, which was redefined as a complete excision in this study.

Of the eight cases with incomplete BCC excision not detected by evRCM, histological examination identified six superficial, one sclerodermiform, and one nodular subtype. The tumor remnants extended between 0.3 and 1.3 mm deep into the dermis. The sclerodermiform BCC could be reliably identified retrospectively by the pathologist on the evRCM image. In five cases, the pathologist could not identify any residuals on the evRCM images, even with knowledge of the histological findings, and in two cases, the findings remained unclear retrospectively. The epidermis was well captured on two evRCM images, moderately captured on two, and poorly captured on four. Taking all findings together, the pathologist estimated the probability that tumor residues were not present in the true surgical margin as high in two cases, possible in four cases, and unlikely in two cases. The pathologist felt very confident in the findings of two evRCM images, moderately confident in two, and uncertain in four.

Within the three cases with a histologically confirmed complete excision, the pathologist could not detect any tumor residues in two cases, and one case was retrospectively assessed as unclear. The visibility of the epidermis was poor in two cases and could not be assessed in the third as it was a basal re-excision.

The pathologist was uncertain because of small focal tumor nests, superficial tumor residues, inflammatory lymphocytic infiltration, and increased skin adnexa. These structures were difficult to assess, especially when the epidermis was not covered.

### 3.3. Management of Discordant Findings

In the eight cases with histologically confirmed residual BCC not identified by evRCM, a defect closure was performed, while a re-excision was performed on the following day in three cases. Histologically, the new surgical margins were tumor-free. Three patients decided against re-excision, preferring local therapy and a follow-up. Two patients had already been discharged when the histological results showing tumor residues became available. A re-excision was carried out during a second inpatient stay after monitoring by OCT.

Three cases were determined as complete excision histologically but inconclusive by evRCM. In one, a small lateral re-excision was performed; in another, a compensatory triangle was removed in the suspicious area; and in the third, a basal re-excision was performed.

### 3.4. Impact on the Length of Hospitalization

With the perioperative use of evRCM, a re-excision or defect closure could be decided upon and performed during the same surgical session without waiting till the following working day for the histological findings, making early discharge possible. Therefore, the hospital stay was shortened by one day on normal working days, two before public holidays, and three on Fridays before the weekend. Overall, the hospital stay of the 90 analyzed patients was shortened by 113 days. In two cases, an additional hospital stay, totaling six days, was necessary due to R1 situations.

## 4. Discussion

evRCM assessment of re-excised tumors showed high specificity but lower sensitivity than in previous studies on surgical margin assessment with evRCM (20% vs. 73.6–94.0%) [28,29,30,31,32,33]. Tumor residuals were diagnosed in two of the ten histologically confirmed incomplete excisions in our study using evRCM. Several factors could explain this sensitivity disparity. In some of the studies achieving high sensitivity, the assessments were not carried out under routine clinical conditions so that sample positioning could be corrected for optimized imaging [28]. Peters et al. [32], Longo et al. [33], and Grupp et al. [28] have also compared evRCM with histopathological paraffin slices under routine clinical conditions. They also reported lower sensitivities between 73.0% and 79.8% compared to higher sensitivities under research conditions of 96.6% [31].

**Figure 2:**
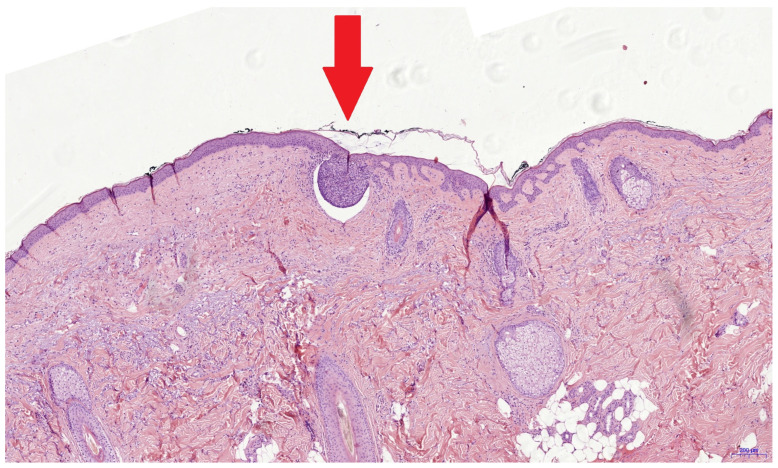
This histological image shows a superficial BCC nest with a diameter of 0.3 mm (red arrow). It is possible that such tumor remnants are not present in the true surgical margin and cannot be seen using evRCM. The histological findings might be false positive due to sectioning of the re-excised specimen.

It was reported that investigators only needed a two-day introduction to confidently use the evRCM device [28]. Safe evRCM assessment might require more time for cutaneous surgeons due to their limited histologic experience. The low rate of positive surgical margins (n = 10) is a limiting factor of this study. In contrast to previous studies, we only analyzed re-excisions following an incomplete primary excision. Accordingly, only a small tumor residue could be expected in the surgical margins, making assessment more difficult. During the pathology processing, the re-excised tissue is placed on the outer edge, cast in paraffin, and sectioned from the outside until a continuous tissue section completely covered by the epidermis can be produced, losing the outer edges in the process. Histological assessment is performed on a section from the inside of the re-excised specimen. Therefore, an incomplete excision may be found in the pathological assessment even though the tumor was completely removed (Figure 2). After the pathologist’s retrospective assessment of the pathological and evRCM findings and a further assessment of the medical history, this situation was possible in four of eight cases and probable in two. Only one false negative incomplete excision could be confirmed retrospectively by the pathologist using the evRCM images. The tumor could be histologically confirmed in two cases in a later re-excision. Consequently, how many of the ten cases were completely removed remains unclear, so the sensitivity can range between 0.2 and 0.8.

**Figure 3:**
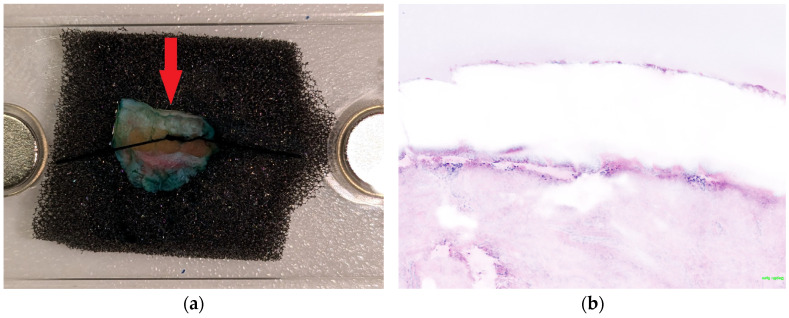
The epidermis often tilts inwards or outwards when clamped between the glass panes. (**a**) The bright stripe marked by the red arrow is the inward-tilted epidermis. Consequently, the epidermis is barely visible in the evRCM image (**b**), and the subepidermal areas might be obscured.

Residual NMSC tumor parts in the lateral surgical margin, especially BCC, are often found superficially, directly on the underside of the epidermis, and have a limited penetration depth, making epidermis visibility essential for re-excision diagnosis. Visualizing the epidermis completely by evRCM is difficult, and its assessment requires experience. While the epidermis is stiff, the dermis and subcutaneous fat tissue are softer and more flexible. Once the tissue is clamped between the glass panes, the laterally overlying epidermis often tilts inwards or outwards and can either not be seen at all or obscure parts of the sub-epidermal dermis, crucial for superficial tumor remnant diagnosis (Figure 3). This effect is intensified if the post-excise is cut round or unevenly. Fixing the tissue with a sponge, molded plasticine, or a splint could help protect the exact position of the epidermis. Moreover, the position under the cover glass can be corrected with a scalpel. Despite these methods, complete visualization of the epidermis is often impossible. In this study, the epidermis was moderately or poorly visible in six of the eight cases with a false-negative diagnosis of tumor-free surgical margins. Limited image quality due to air bubbles and rolling or incompletely adherent epidermis has also been reported as a problem with evRCM [27,28,32,37]. Preparation requires some time and experience. The operation is significantly extended if the operating surgeon also prepares, acquires the images, and diagnoses using evRCM. If possible, an experienced physician assistant should carry out tissue preparation and evRCM imaging.

**Figure 4:**
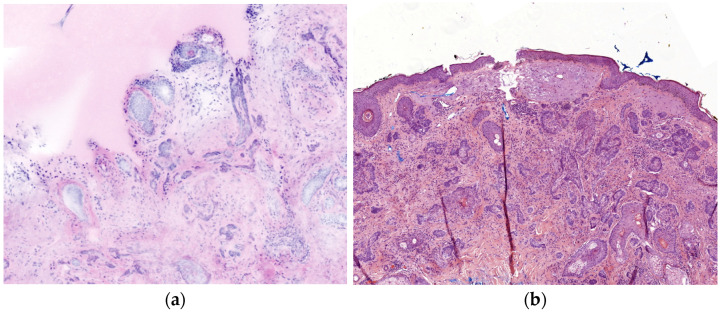
Comparison of a sclerodermiform BCC between evRCM and pathology. (**a**) Due to the lower contrast and the lack of epidermis, it is difficult to distinguish between sclerodermiform tumor nests and skin adnexal structures in the evRCM images. (**b**) The sclerodermiform BCC is easy to recognize on the histological slide.

Digital staining in evRCM produces HE-like images, which look familiar from histology. Previous experience with other imaging techniques (e.g., RCM, LC-OCT, OCT) is helpful but is insufficient to reliably diagnose using evRCM. In vivo RCM and LC-OCT are used to examine the upper 200 µm of the skin, sometimes in horizontal sections. Close cooperation with a pathologist and correlation with the histology slides is recommended to reliably diagnose deeper parts of the dermis. In this study, the pathologist discovered one of the eight false-negative cases retrospectively, while the cutaneous surgeon made the initial diagnosis. Despite digital staining, histological slides and evRCM images look different. In particular, pathologists need to learn how to interpret evRCM images due to color differences in skin adnexal structures and lymphocytic infiltration, the frequent lack of reference to the epidermis, and artifacts (Figure 4). Grupp et al. [28] reported that sebaceous glands, inflammatory lymphocytic infiltration, epidermal cones, or hair follicles were reasons for false positive results.

Within the study population, 27 (28.7%) skin tumors were located on the nose. The localization of skin cancer in specific facial aesthetic units has been shown in multiple studies to be an independent risk factor for recurrence [38]. The central face with the nose is an area with a high risk of recurrence for basal cell carcinoma [39]. The high proportion of skin tumors on the nose in this study may have influenced the results. Other risk factors for recurrences are certain histological subtypes of basal cell carcinoma or neuro-vascular infiltration of squamous cell carcinoma [40].

Due to slightly different levels in the re-excised tissue, examined using evRCM and pathology, and the discussed limitations of evRCM, it is to be expected that discordant findings will occur regularly, even in experienced surgical teams. A standardized procedure should be defined for these cases. Patients should be informed before using evRCM that discordant results can occur and that incomplete excisions can potentially be overlooked in evRCM. In the event of an incomplete excision, the patient should be advised to undergo re-excision. If the patient refuses, local therapy and close follow-up can be carried out to recognize a pseudo-recurrence at an early stage. OCT, in vivo RCM, or LC-OCT can effectively support the follow-up checks.

Using evRCM, the surgical margins can be assessed perioperatively, and the surgical consequence can be implemented directly. In-hospital stays can be greatly shortened, especially in hospitals where 3D histology with overnight tissue processing is used. In this study, patients examined with evRCM were discharged on average 1.3 days earlier. Shortened hospital stays reduce costs, decrease frequent complications associated with hospital stay in older adult patients, such as nosocomial infections or immobilization, and increase patient satisfaction.

## 5. Conclusions

evRCM can be used to assess the surgical margins of re-excised NMSCs, reducing in-hospital stay duration. The pathology and evRCM findings may differ because evRCM has various limitations and assesses the true surgical margins of the incision, while pathology assesses slightly deeper tissue levels. Good communication is necessary to ensure making the right decision for the patient in these cases. Tissue sample preparation for evRCM requires time and experience and should, if possible, be carried out by a medical-technical assistant in parallel to the surgery. Both dermatologists and pathologists must be trained to assess evRCM images. Close cooperation between the two specialties could help reduce the number of discordant findings.

## Figures and Tables

**Table 1:** Patient and tumor characteristics.

**Patient Characteristics**	
Patients (*n*)	90
Male, *n* (%)	47 (52.2)
Female, *n* (%)	43 (47.8)
Age, mean ± SD (range)	78.5 ± 10.3 (38–94) years
**Tumor Characteristics**	
Excision (*n*)	94
Basal cell carcinoma, *n* (%)	82 (87.2)
Nodular BCC (*n*)	50
Sclerodermiform BCC (*n*)	21
Superficial BCC (*n*)	14
Micronodular BCC (*n*)	9
Cystic BCC (*n*)	4
Metatypical BCC (*n*)	1
BCC tumor thickness, mean ± SD (range)	2.1 ± 1.4 (0.3–6.2) mm
BCC infiltration level, mean ± SD (range)	4.1 ± 0.4 (2–5)
Squamous cell carcinoma, *n* (%)	11 (11.7%)
SCC tumor thickness, mean ± SD (range)	3.0 ± 2.0 (1.5–6.5) mm
SCC infiltration level, mean ± SD (range)	4.5 ± 0.5 (4–5)
SCC grade, mean ± SD (range)	2.0 ± 0.4 (1–3)
Bowen’s disease, *n* (%)	1 (1.1%)

**Table 2:** Cross tabulation showing the concordance of the evRCM and histological findings and the sensitivity, specificity, and diagnostic accuracy of evRCM.

	Histology—R1	Histology—R0	Total
evRCM—R1	2	3	5
evRCM—R0	8	81	89
Total	10	84	94
Sensitivity	0.20 (95% CI, 0.06–0.51)		
Specificity	0.96 (95% CI, 0.90–0.99)		
Accuracy	0.88 (95% CI, 0.80–0.93)		

evRCM: ex vivo confocal laser scanning microscopy; R1: tumor remnants in the surgical margin; R0: no evidence of a tumor in the surgical margin.

## Data Availability

The data presented in this study are available on request from the corresponding author. Due to the evaluation of the data in accordance with §29 (3) of the Saxon Hospital Act, data may only be used by members of TU Dresden for scientific research. To use the data outside the TU Dresden, a new ethics application is required.
